# Optimization of Hydrophobic Group Structures Covalently Incorporated into Gelatin Hydrogels for Efficient Adsorption and Release of Hydrophobic Drugs

**DOI:** 10.3390/gels12100941

**Published:** 2026-10-21

**Authors:** Ko Nishimura, Takanori Matsumoto, Kazunori Kanamaru, Saki Kobaru, Takeshi Ishikawa, Yuna Tatsumi, Yusuke Shimoyama, Masahiro Yoshida, Takayuki Takei

**Affiliations:** 1 Department of Chemical Engineering, Graduate School of Science and Engineering, Kagoshima University, 1-21-40 Korimoto, Kagoshima 890-0065, Japan; k7787358@kadai.jp (K.N.); inamine@eng.kagoshima-u.ac.jp (S.K.); myoshida@cen.kagoshima-u.ac.jp (M.Y.); 2 Department of Chemistry, Biotechnology, and Chemical Engineering, Graduate School of Science and Engineering, Kagoshima University, 1-21-40 Korimoto, Kagoshima 890-0065, Japan; ishi@cb.kagoshima-u.ac.jp; 3 Department of Chemical Science and Engineering, Institute of Science Tokyo, 2-12-1 Ookayama, Tokyo 152-8550, Japanshimoyama.y.37ad@m.isct.ac.jp (Y.S.)

**Keywords:** gelatin, hydrogel, hydrophobic interaction, hydrophobic drug, sustained release

## Abstract

Hydrophobically modified gelatin (HMG) hydrogels are promising proof-of-concept drug delivery carriers. However, the structural effects of incorporated hydrophobic groups remain systematically unexamined. Here, twelve linear fatty and aromatic hydrophobic groups with varying carbon numbers were incorporated into gelatin to identify optimal structures for hydrophobic drug loading. Adsorption behaviors of three drug models were quantitatively evaluated per mole of incorporated carbon atoms. Linear fatty aldehydes with 11 and 12 carbon atoms (F-CHO-C11 and C12) achieved maximum adsorption efficiency, with equilibrium adsorption capacities of $0.35\times 10^{-3}$ and $0.34\times 10^{-3}$ mol-rhodamine B/mol-carbon atoms, respectively. Thermodynamic calculations (COSMO-SAC) and quantum chemical simulations (Gaussian MP2) supported the idea that longer alkyl chains enhance affinity, yielding higher interaction energy (−11.8kcal/mol for C12 vs. −5.73kcal/mol for C4). Furthermore, F-CHO-C12 HMG hydrogel achieved a substantial dexamethasone loading capacity of approximately $2.8\times 10^{-6}$ mol/g-dry polymer (unmodified gelatin showed negligible loading $<0.08\times 10^{-6}$ mol/g-dry polymer) and sustained release at 37 °C (∼95% cumulative release without initial burst). These findings establish quantitative design guidelines for hydrophobically modified polymer hydrogels.

## 1. Introduction

Hydrogels consist of three-dimensional polymeric networks that retain large amounts of water. The high water content of hydrogels is similar to that of living tissue, making hydrogels highly biocompatible [1,2,3,4]. The high biocompatibility has expanded the use of hydrogels as carriers for sustained release of drugs [5,6]. 

Drugs can be divided into two different types: hydrophilic and hydrophobic drugs. Hydrogels composed of hydrophilic polymers are suitable as carriers for hydrophilic drugs but not for hydrophobic drugs. This is because the low affinity between hydrophilic polymer networks and hydrophobic drugs often leads to an initial burst release [7,8,9]. Hydrophobic modification of hydrophilic polymers has been reported to increase the affinity between polymers and hydrophobic drugs [9,10,11]. Hydrophobic drugs can adsorb onto hydrophobic portions of the polymers by hydrophobic interactions, thereby avoiding the burst release. Optimizing the structure of hydrophobic groups is of paramount importance for sustained release of the drugs.

Gelatin is a hydrophilic protein obtained by acid or alkaline hydrolysis of collagen. The protein has been widely used as a carrier of various drugs because of its excellent biocompatibility and biodegradability [12,13,14,15]. Gelatin hydrogels are prepared by cooling a gelatin aqueous solution as a result of the molecules forming a triple helix [16,17,18]. However, the hydrogels dissolve at a physiological temperature owing to the disruption of the triple helix. To obtain a stable gelatin hydrogel at the physiological temperature, gelatin molecules have been cross-linked with cytotoxic chemical cross-linkers such as glutaraldehyde [19,20]. However, such hydrogels prepared using cytotoxic chemicals are not suitable for medical applications. On the other hand, we have prepared a hydrogel composed of hydrophobically modified gelatin (HMG) that is stable at physiological temperature [21]. The main cross-linking points of the hydrogel would be formed by hydrophobic interactions between the hydrophobic groups of HMG molecules. We have shown that a hydrophobic drug model adsorbed to the HMG hydrogels by hydrophobic interactions. We also showed that the adsorbed hydrophobic molecules were gradually released. However, the hydrophobic modification in our previous work was limited solely to dodecyl (C12) linear fatty chains.

**Figure 1:**
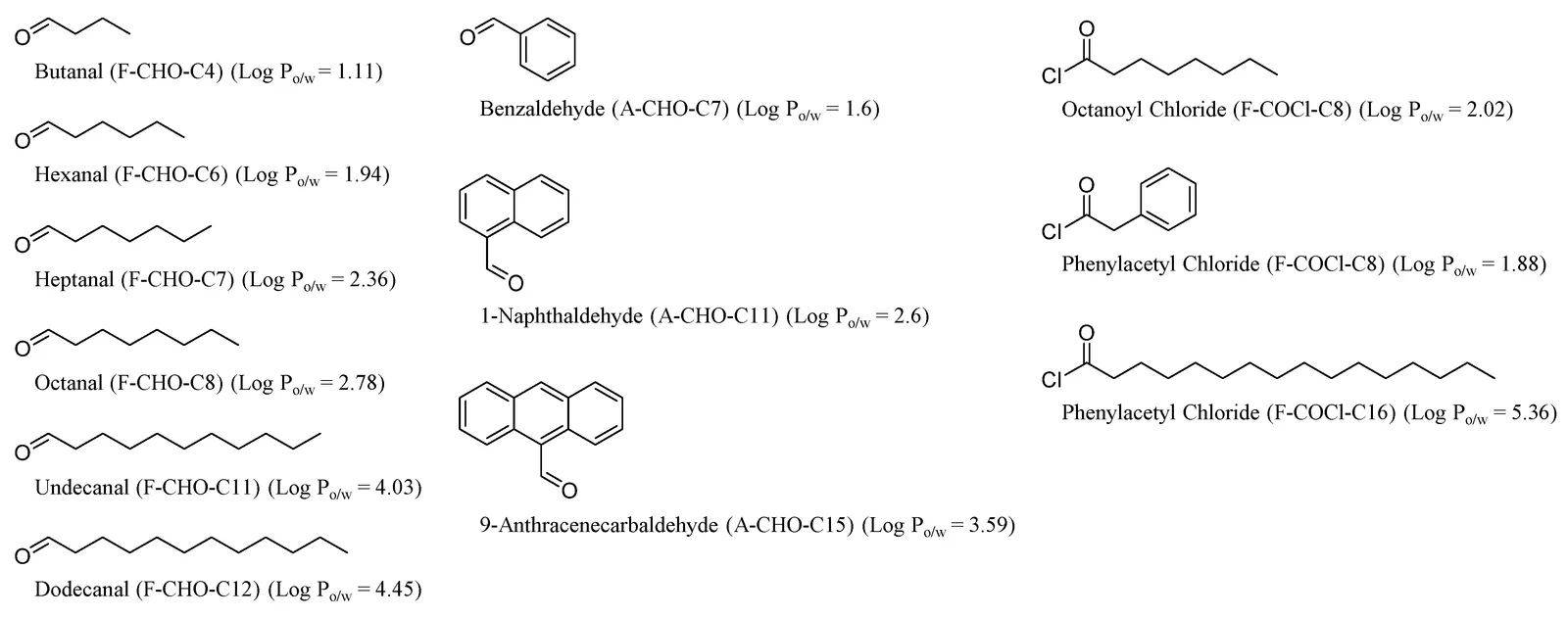
Chemical structures of hydrophobic groups incorporated into gelatin. The first, second, and third components of each abbreviation represent linear fatty (F) or aromatic (A), the reactive functional group (aldehyde (CHO) or acyl groups (COCl)), and the number of carbon atoms, respectively. The octanol–water partition coefficients (Log P_o/w_) were calculated according to Section 4.5.

**Figure 2:**
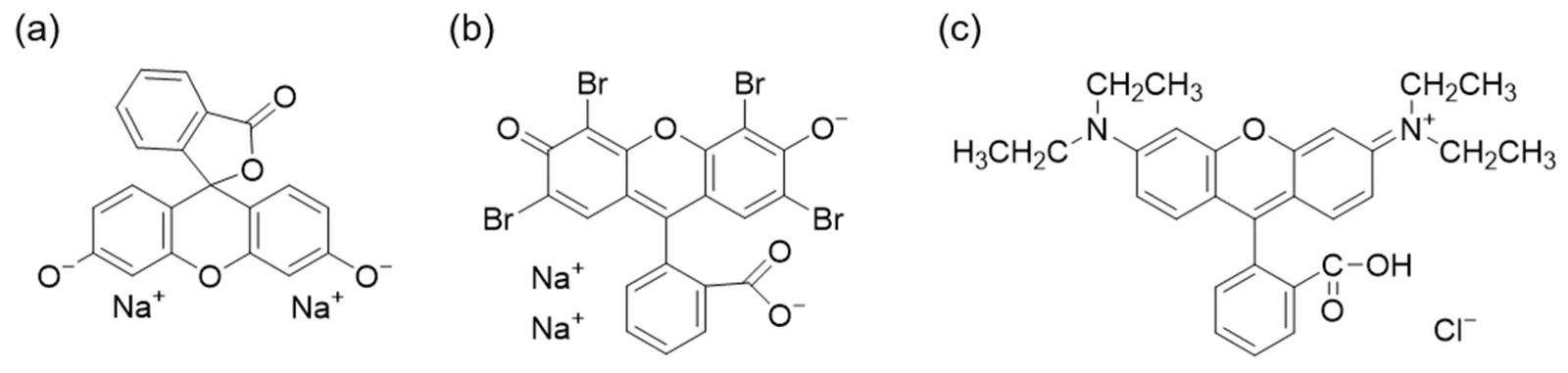
Chemical structures of uranine (**a**), eosin (**b**) and rhodamine-B (**c**) as hydrophobic drug models. Log P_o/w_ values of these drug models at neutral pH were −0.99 [23], −0.18 [24] and 2.30 [25], respectively.

In this study, we aimed to identify the optimal structure of hydrophobic groups incorporated into gelatin as carriers of hydrophobic drugs. Herein, the optimal structure refers to the hydrophobic group that yields a high adsorption amount of hydrophobic drugs per mole of carbon atoms contained in the incorporated hydrophobic groups of HMG. To achieve this goal, we incorporated twelve kinds of hydrophobic groups with different structures and carbon atom number into gelatin (Figure 1). Next, three hydrophobic drug models with different hydrophobicity were used to examine their adsorption behavior on HMG hydrogels (Figure 2). Furthermore, the differences in adsorption amounts among the hydrophobic groups were analyzed using their activity coefficients calculated by the conductor-like screening model-segment activity coefficient method (COSMO-SAC) [22] aided by a quantum chemical calculation. Finally, the HMG with the optimized hydrophobic groups was used to evaluate the adsorption and release of dexamethasone, a hydrophobic anti-inflammatory drug.

## 2. Results and Discussion

### 2.1. Synthesis of HMG

**Figure 3:**
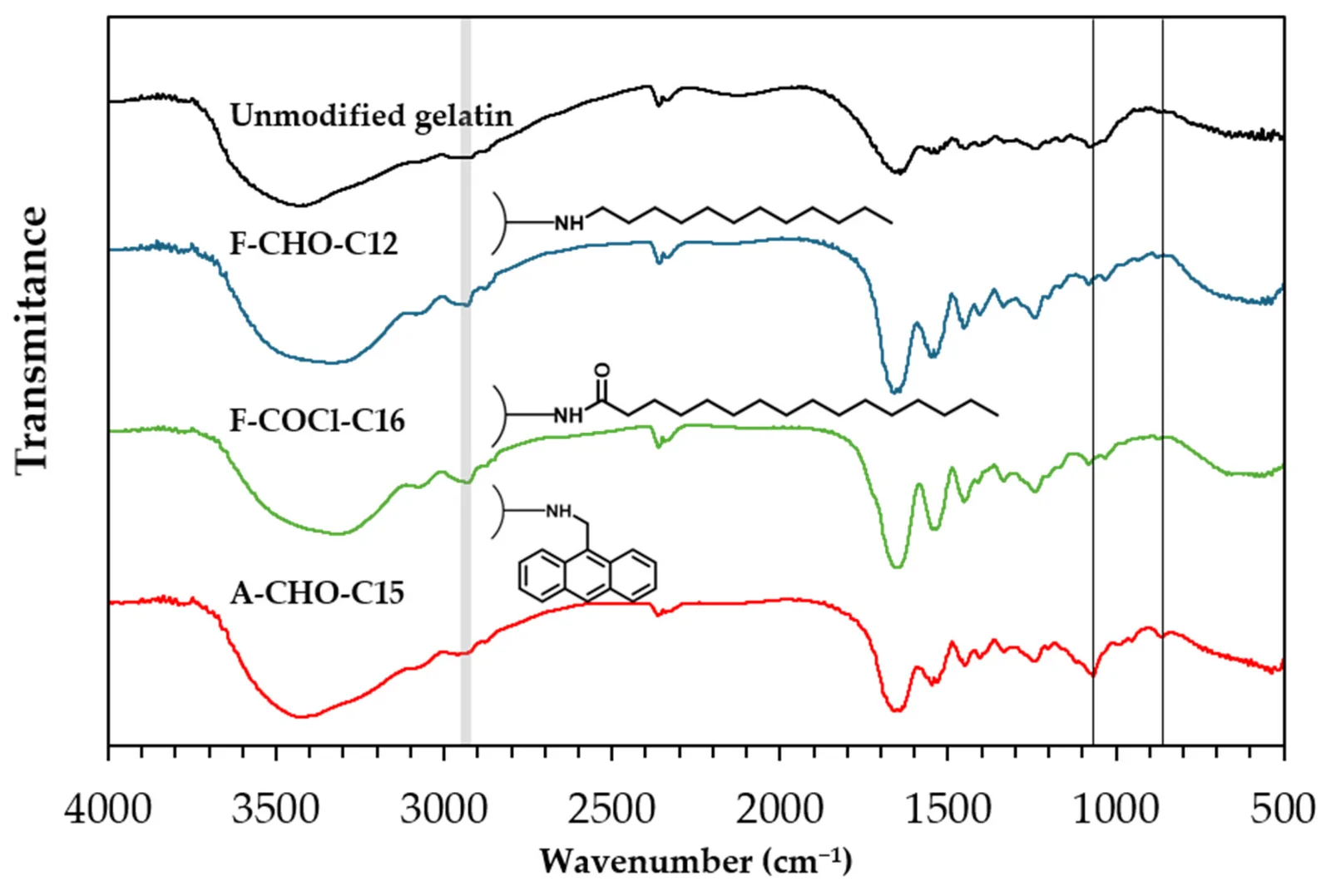
FT-IR spectra of unmodified gelatin and HMG.

In this study, we aimed to investigate how structure and the carbon number of hydrophobic groups incorporated into gelatin affected the adsorption amount of hydrophobic drugs onto HMG hydrogel. It is also important to investigate how differences in the chemical bonds at the modified sites after the chemical reaction between the reactive groups of the hydrophobic groups and amino groups of gelatin affect the adsorption amount of hydrophobic drugs. To clarify the influence of the structure of hydrophobic groups, we compared two types of hydrophobic groups: linear fatty and aromatic. In addition, to examine the influence of the carbon number of hydrophobic groups, we compared hydrophobic groups with different carbon numbers. Finally, to examine the influence of the chemical bond at the modified sites, we adopted aldehydes and acyl chlorides as reactive groups of hydrophobic groups. The hydrophobic groups used in this study are summarized in Figure 1 and Table 1. The carbon numbers shown in the names of each HMG in Figure 1 and Table 1 contain a carbon atom in the reactive group (aldehyde and acyl chloride). Hydrophobic groups with aldehyde groups and acyl chloride groups were incorporated into gelatin according to the chemical reactions shown in Figures S1 and S2, respectively [21,26,27]. To confirm the incorporation of hydrophobic groups, FT-IR (Fourier transform infrared spectroscopy) measurements were carried out. Figure 3 shows the FT-IR spectra of unmodified gelatin and HMG with either F-CHO-C12, F-COCl-C16 or A-CHO-C15 as a representative linear fatty aldehyde, linear fatty acyl chloride or aromatic groups, respectively. Compared with unmodified gelatin, HMG with linear fatty groups (F-CHO-C12 and F-COCl-C16) showed a peak derived from –CH_2_ at around 2930–2945 cm^−1^ [28]. These results provide qualitative evidence for the incorporation of linear fatty groups into gelatin. For the HMG with aromatic groups (A-CHO-C15), peaks attributable to aromatic C–H out-of-plane bending were observed at 864 cm^−1^. In addition, a peak attributable to aromatic C–H in-plane bending was observed at 1067 cm^−1^. These results provide qualitative evidence for the incorporation of aromatic groups into gelatin. The degrees of substitution of each hydrophobic group were unified to approximately 40% by changing the feeding molar ratios to the free amino groups of gelatin (Table 1).

**Figure 4:**
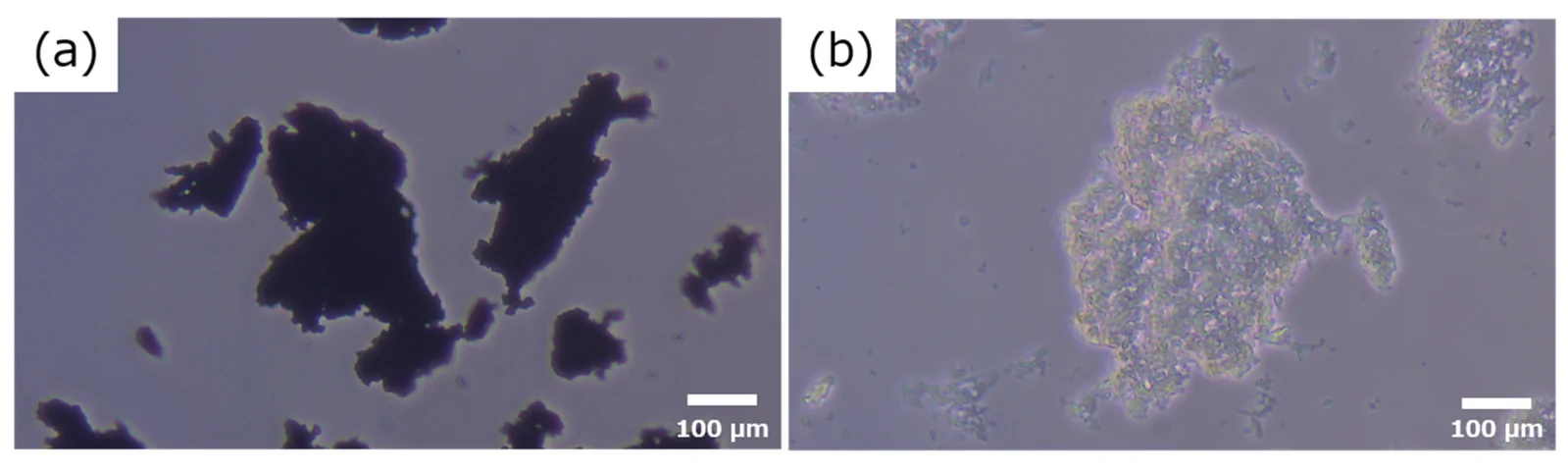
Photos of HMG microparticles prepared using F-CHO-C4 before (**a**) and after (**b**) swelling in PBS(-).

**Figure 5:**
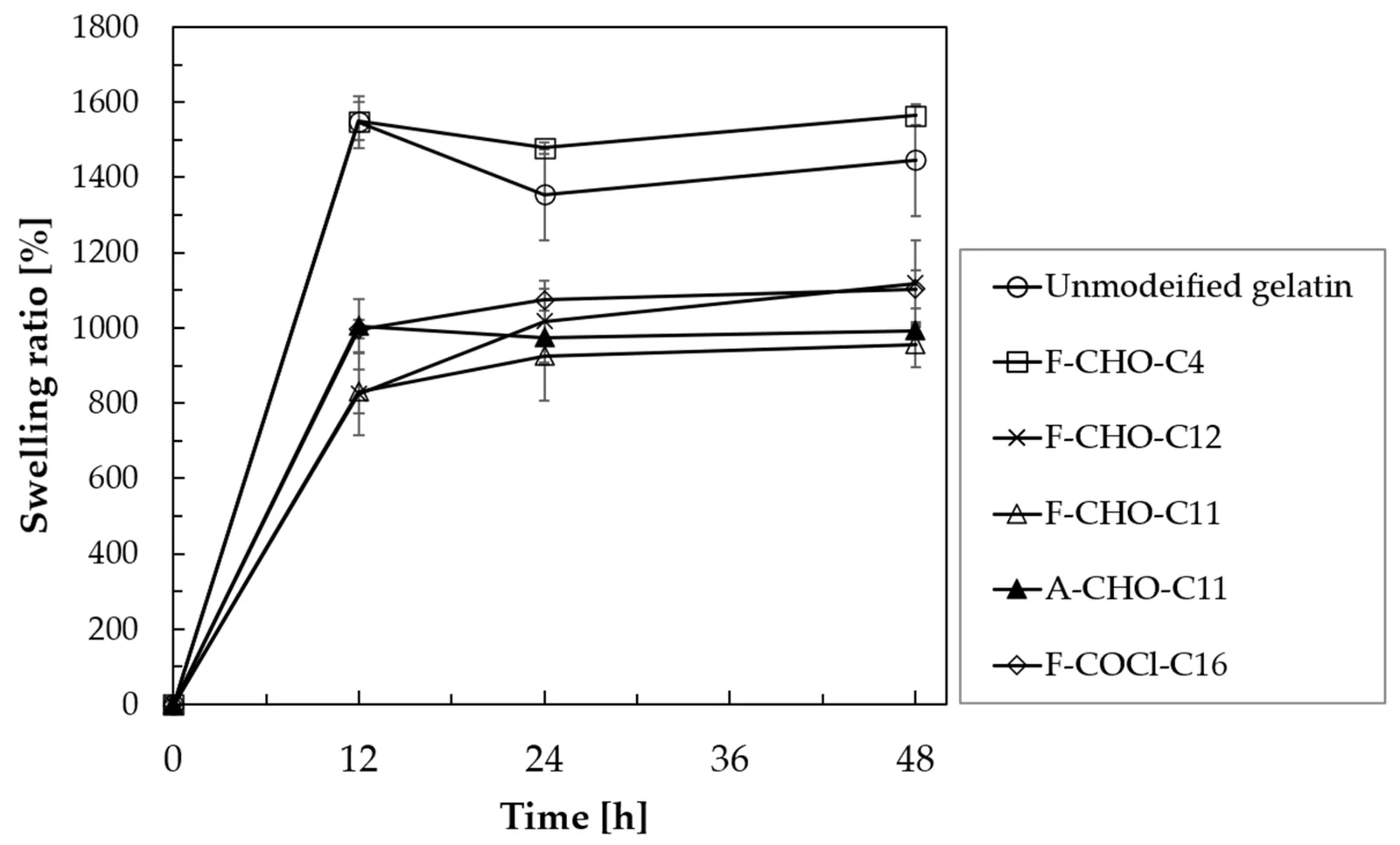
Swelling behavior of HMG (*n* = 3).

Solid unmodified gelatin and HMG particles (<1 mm in diameter) were immersed in phosphate-buffered saline without calcium and magnesium ions (PBS(-)) at 4 °C for more than 20 min to hydrate the particles (Figure 4 and Figure S3). As a result of this treatment, the particles transmitted light, showing that the particles changed from a solid state into hydrogels. We confirmed that uncrushed HMG dry solids added to PBS(-) in the same manner formed a self-supporting bulk hydrogel. The swelling behavior of HMG particles was evaluated by measuring their swelling ratio (Figure 5). F-CHO-C4 exhibited a swelling ratio comparable to that of unmodified gelatin. In contrast, the HMGs modified with hydrophobic groups containing a larger number of carbon atoms (F-CHO-C11, F-CHO-C12, A-CHO-C11, and F-COCl-C16) showed lower swelling ratios than unmodified gelatin. Furthermore, no marked difference in swelling ratio was observed between A-CHO-C11, bearing an aromatic group, and F-CHO-C11, bearing a linear alkyl group. These results suggest that the introduction of hydrophobic groups with a larger number of carbon atoms reduced the amount of water incorporated into the HMG network, thereby forming a more hydrophobic internal environment. These hydrogel particle samples were used in the subsequent experiments. 

### 2.2. Adsorption of Hydrophobic Drug Models on HMG Hydrogel

**Figure 6:**
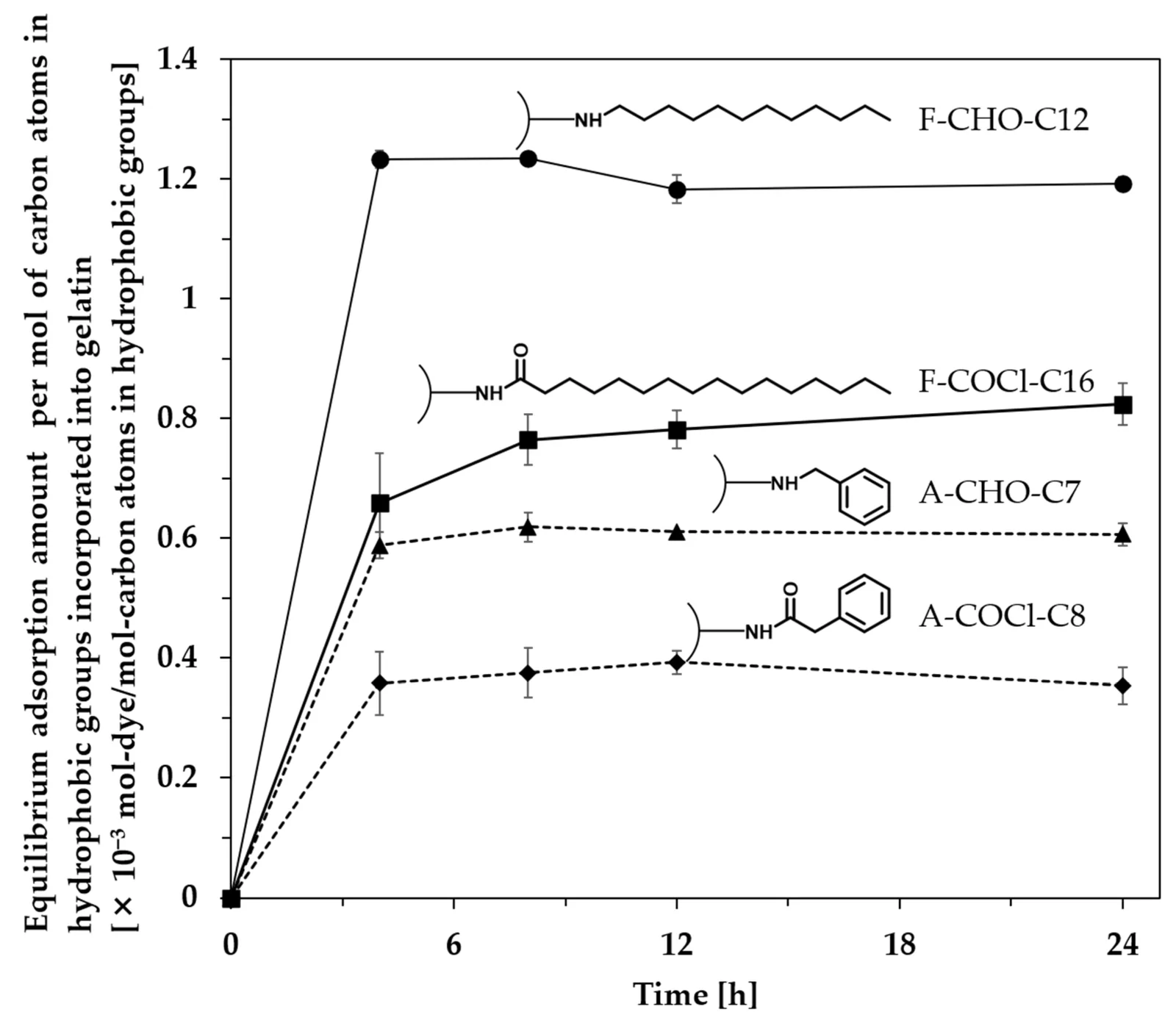
Adsorption profile of eosin Y on HMG hydrogel particles with A-CHO-C7, A-COCl-C8, F-CHO-C12 or F-COCl-C16 at 4 °C (*n* = 3).

**Figure 7:**
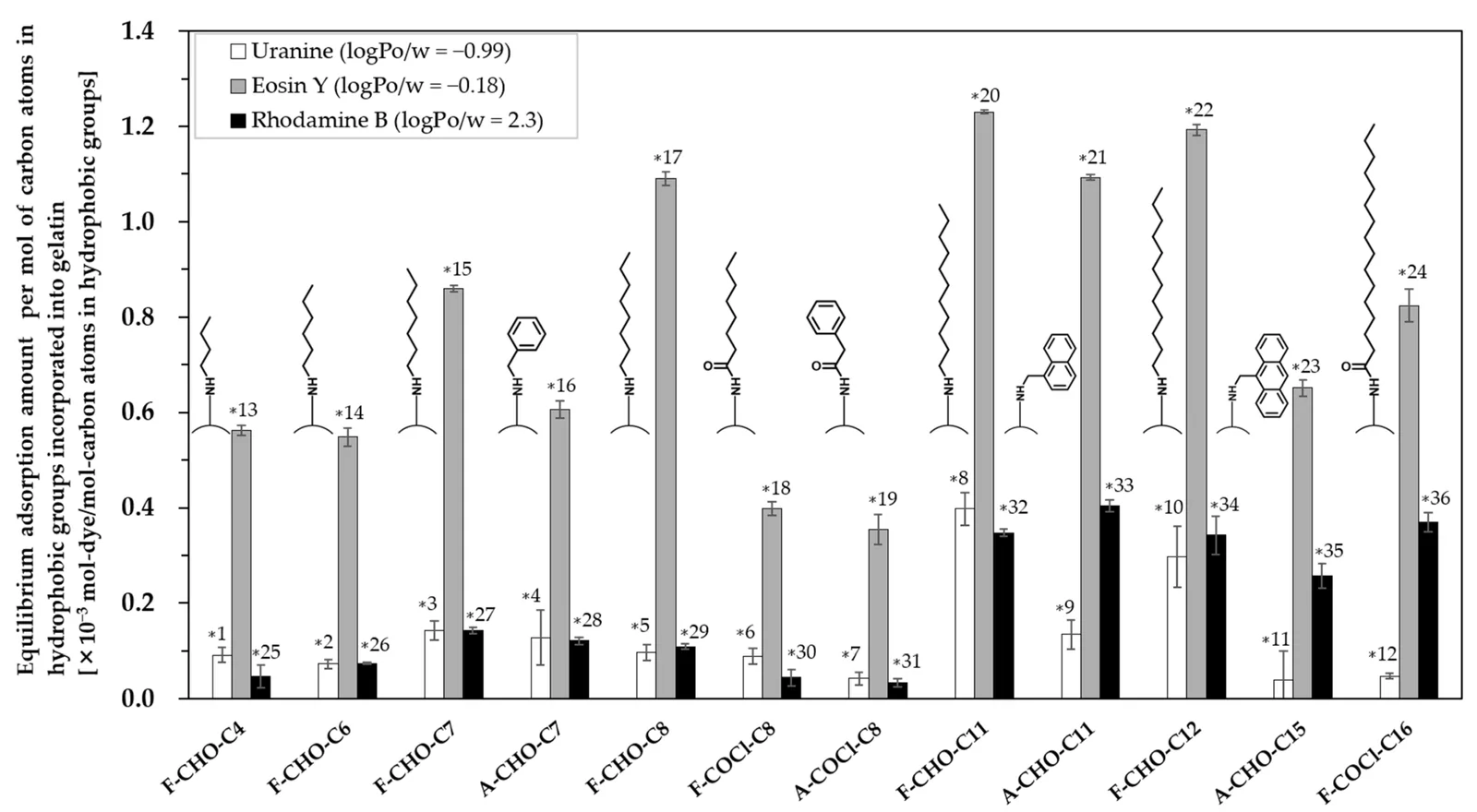
Adsorption amount of hydrophobic drug models on HMG hydrogel particles at 4 °C (*n* = 3), *^1–^*^7,^ *^9,^ *^11,^ *^12^ *p* < 0.05 vs. F-CHO-C11, F-CHO-C12, *^8^ *p* < 0.05 vs. all samples except for F-CHO-C12, *^10^ *p* < 0.05 vs. all samples except for F-CHO-C11, *^13,^ *^14^ *p* < 0.05 vs. all samples except for F-CHO-C6, A-CHO-C7, *^15^ *p* < 0.05 vs. all samples except for F-COCl-C16, *^16^ *p* < 0.05 vs. all samples except for F-CHO-C4, F-CHO-C6, F-CHO-C15, *^17^ *p* < 0.05 vs. all samples except for A-CHO-C11, *^18^ *p* < 0.05 vs. all samples except for A-COCl-C8, *^19^ *p* < 0.05 vs. all samples except for F-COCl-C8, *^20^ *p* < 0.05 vs. all samples except for F-CHO-C12, *^21^ *p* < 0.05 vs. all samples except for F-CHO-C8, *^22^ *p* < 0.05 vs. all samples except for F-CHO-C11, *^23^ *p* < 0.05 vs. all samples except for A-CHO-C7, *^24^ *p* < 0.05 vs. all samples except for F-CHO-C7, *^25^ *p* < 0.05 vs. all samples except for F-CHO-C6, F-CHO-C8, F-COCl-C8, A-COCl-C8, *^26^ *p* < 0.05 vs. all samples except for F-CHO-C4, A-CHO-C7, F-CHO-C8, F-COCl-C8, A-COCl-C8, *^27^ *p* < 0.05 vs. all samples except for A-CHO-C7, F-CHO-C8, *^28^ *p* < 0.05 vs. all samples except for F-CHO-C6, F-CHO-C7, F-CHO-C8, *^29^ *p* < 0.05 vs. all samples except for F-CHO-C4, F-CHO-C6, F-CHO-C7, A-CHO-C7, F-COCl-C8, *^30^ *p* < 0.05 vs. all samples except for F-CHO-C4, F-CHO-C6, F-CHO-C8, A-COCl-C8, *^31^ *p* < 0.05 vs. all samples except for F-CHO-C4, F-CHO-C6, F-COCl-C8, *^32^ *p* < 0.05 vs. all samples except for A-CHO-C11, F-CHO-C12, F-COCl-C16, *^33^ *p* < 0.05 vs. all samples except for F-CHO-C11, F-CHO-C12, F-COCl-C16, *^34^ *p* < 0.05 vs. all samples except for F-CHO-C11, A-CHO-C11, F-COCl-C16, *^35^ *p* < 0.05 vs. all samples, *^36^ *p* < 0.05 vs. all samples except for F-CHO-C11, A-CHO-C11, F-CHO-C12.

In this study, we determined the adsorption amount of drug models per mole of carbon atoms contained in the incorporated hydrophobic groups of HMG hydrogel (not per unit weight of HMG). To fairly compare the adsorption amounts, it is necessary to make the total number of moles of carbon atoms in the incorporated hydrophobic groups added to the drug model aqueous solutions the same for all samples. This means that, for example, when HMG hydrogel particles with 1 mol of C12 groups are added to the drug model aqueous solution, HMG with C4 groups must be added at 3 mol to maintain an equivalent total carbon atom amount (Figure S4). In this study, HMG hydrogel particles were added to 4 mL of drug model aqueous solution so that the total number of moles of carbon atoms in the hydrophobic groups contained in the particles became 7.5 × 10^−5^ mol in the solution and shaken until adsorption equilibrium was reached. Figure 6 shows the adsorption behavior of eosin Y onto HMG hydrogel particles with A-CHO-C7, A-COCl-C8, F-CHO-C12 and F-COCl-C16 as representative HMG samples. HMG with hydrophobic groups other than those mentioned above also showed similar adsorption behavior of eosin Y. In addition, similar behavior was observed in the case of uranine and rhodamine B for all samples. These results show that adsorption of all drug models reached equilibrium within 24 h. Therefore, the adsorption amounts of drug models after 24 h of incubation were compared for each type of HMG hydrogel particle (Figure 7).

Among HMGs possessing hydrophobic groups with carbon numbers of 8 or less, although some statistically significant differences were observed in the adsorption amounts of uranine and rhodamine B, the variations in adsorption capacity were notably larger for eosin Y. Based on these results, we first clarified the influence of the hydrophilic groups at the chemically modified sites on the adsorption amounts by comparing F-CHO-C8 with F-COCl-C8 using eosin Y. F-COCl-C8, which forms carbonyl groups at the modified sites, showed a lower adsorption amount of eosin Y per mole of incorporated carbon atoms than F-CHO-C8. These results are presumably so because the hydrophobicity of the incorporated hydrophobic groups was reduced by the hydrophilic carbonyl groups, as suggested by Log P_o/w_ of hydrophobic groups after chemical reactions to incorporate them into gelatin (Log P_o/w_ = 2.78 (F-CHO-C8) and 2.02 (F-COCl-C8)) (see Section 4.5). These results indicate that aldehyde groups are more suitable than acyl chloride groups as reactive functional groups of hydrophobic groups.

Second, we clarified the influence of the carbon number of aromatic hydrophobic groups on the adsorption amounts by comparing A-CHO-C7, A-CHO-C11 and A-CHO-C15. The reactive groups of the hydrophobic groups were unified to aldehyde groups. For the most hydrophilic drug model, uranine, there was no significant difference in the adsorption amounts among them. The adsorption amounts of eosin Y and rhodamine B onto HMG with A-CHO-C11 were larger than those onto HMG with A-CHO-C7. These results are presumably so because the naphthalene ring (A-CHO-C11, Log P_o/w_ = 2.60) is more hydrophobic than the benzene ring (A-CHO-C7, Log P_o/w_ = 1.60). On the other hand, although the anthracene ring (A-CHO-C15, Log P_o/w_ = 3.59) is more hydrophobic than the naphthalene ring, the adsorption amounts of eosin Y and rhodamine B onto HMG with anthracene rings were lower than those onto HMG with naphthalene rings. There are two possible factors responsible for this behavior. One possible factor is that the high hydrophobicity of the anthracene rings causes self-aggregation, which reduces the number of adsorption sites for the drug models. The other possible factor is the effect of steric hindrance. Since the anthracene ring has the same three-ring structure as eosin Y and rhodamine B, steric hindrance between the anthracene rings and the drug models becomes larger than that between the naphthalene rings and the drug models, making adsorption more difficult. From these findings, it was revealed that among the aromatic hydrophobic groups selected in this study, there exists an optimal carbon number (C11, naphthalene ring). 

Third, we clarified the differences between linear fatty and aromatic hydrophobic groups with the same carbon number (F-CHO-C7 vs. A-CHO-C7, F-COCl-C8 vs. A-COCl-C8, and F-CHO-C11 vs. A-CHO-C11). For acyl chlorides (F-COCl-C8 vs. A-COCl-C8), there was no significant difference in the adsorption amounts of the drug models. These results are presumably due to the strong influence of the hydrophilic carbonyl groups at the modified sites as described above. For carbon number 7 (F-CHO-C7 vs. A-CHO-C7), the adsorption amount of eosin Y onto HMG with linear fatty groups (F-CHO-C7) was higher than that onto HMG with aromatic groups (A-CHO-C7). For carbon number 11 (F-CHO-C11 vs. A-CHO-C11), there was no significant difference in the adsorption amounts of rhodamine B. In contrast, for uranine and eosin Y, the adsorption amounts onto HMG with F-CHO-C11 were higher than those onto HMG with A-CHO-C11. These results are presumably so because, as discussed later, these drug models were adsorbed in a manner that allowed them to be taken up into the linear fatty side chains. 

Finally, we clarified how the carbon number of the linear fatty groups (F-CHO-C4 vs. C6 vs. C7 vs. C8 vs. C11 vs. C12, F-COCl-C8 vs. F-COCl-C16) affects the adsorption amounts of the hydrophobic drug models. For linear fatty aldehydes (F-CHO-C4 vs. C6 vs. C7 vs. C8 vs. C11 vs. C12), the adsorption amounts increased as the carbon number increased. The adsorption amounts are also considered to reach their maximum when the linear fatty groups possess 11 or 12 carbon atoms. For linear fatty acyl chlorides (F-COCl-C8 vs. F-COCl-C16), the adsorption amounts increased as the linear chains became longer, even though hydrophilic carbonyl groups were present at the modified sites. These results are presumably so because the longer chains counteracted the influence of the hydrophilic chemical bond at the modified sites. These results indicate that F-CHO-C11 and F-CHO-C12 are suitable hydrophobic groups to be incorporated into gelatin for adsorption of hydrophobic drugs.

**Figure 8:**
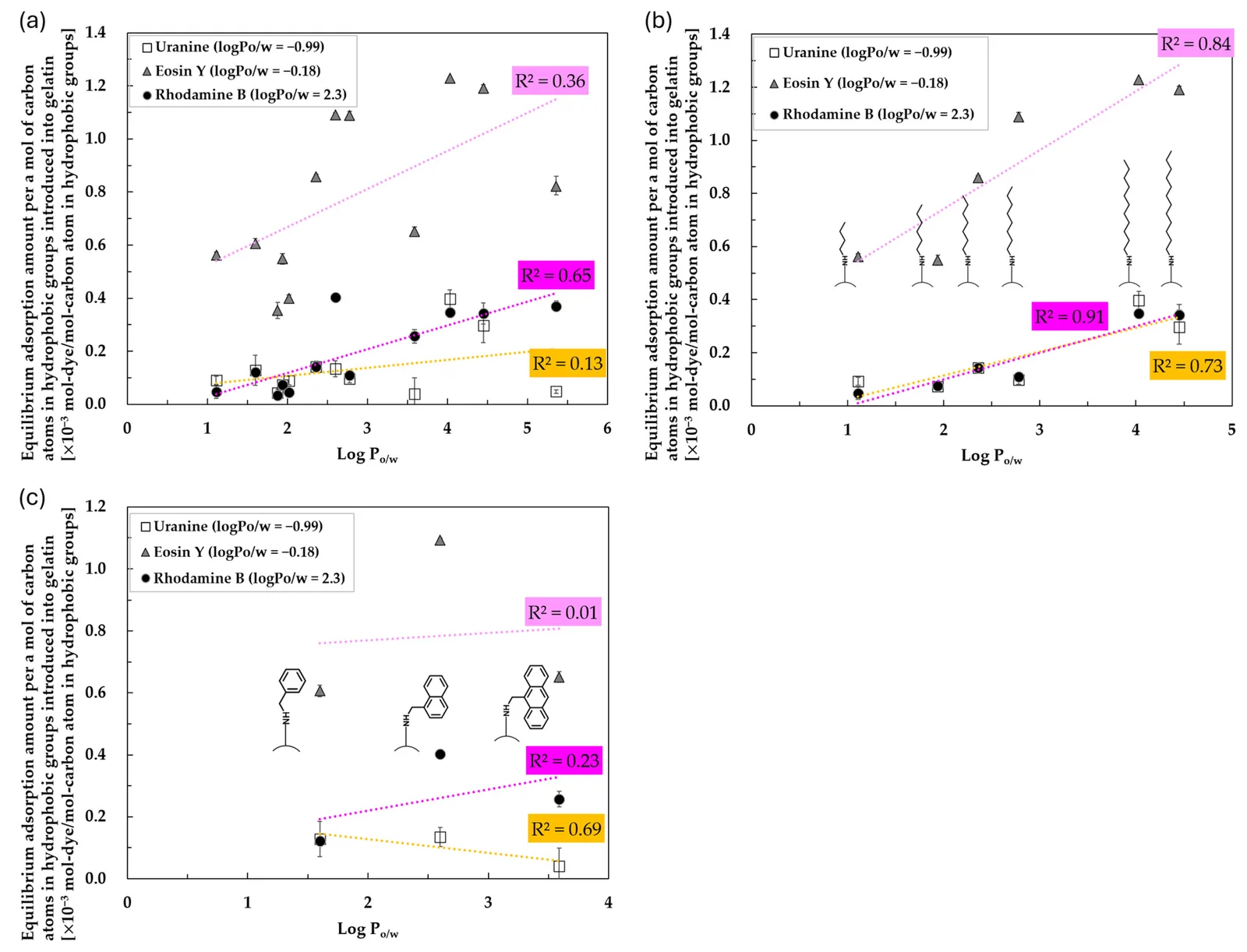
Relationship between Log P_o/w_ of all samples (**a**), linear fatty aldehydes (**b**) or aromatic aldehydes (**c**), and equilibrium adsorption amount.

Figure 8a shows the relationship between the calculated Log P_o/w_ of the incorporated hydrophobic groups and the equilibrium adsorption amount for all samples. Among the model dyes, rhodamine B exhibited the highest positive linear correlation ($R^{2}=0.65$) across all hydrophobic group structures. When the analysis was restricted specifically to linear fatty aldehydes, the positive correlation between Log P_o/w_ and adsorption capacity increased remarkably for all model dyes ($R^{2}=0.73-0.91$; Figure 8b). In contrast, when restricted to aromatic groups, the positive correlation dropped significantly (Figure 8c). These quantitative results demonstrate that for linear fatty groups, calculated hydrophobicity (Log P_o/w_) serves as a primary governing factor for drug adsorption. Conversely, for aromatic groups, simple hydrophobicity alone cannot fully predict adsorption behavior, highlighting the substantial impact of topological features, steric hindrance, and self-aggregation.

### 2.3. Prediction of Adsorption Amount by Calculations

**Figure 9:**
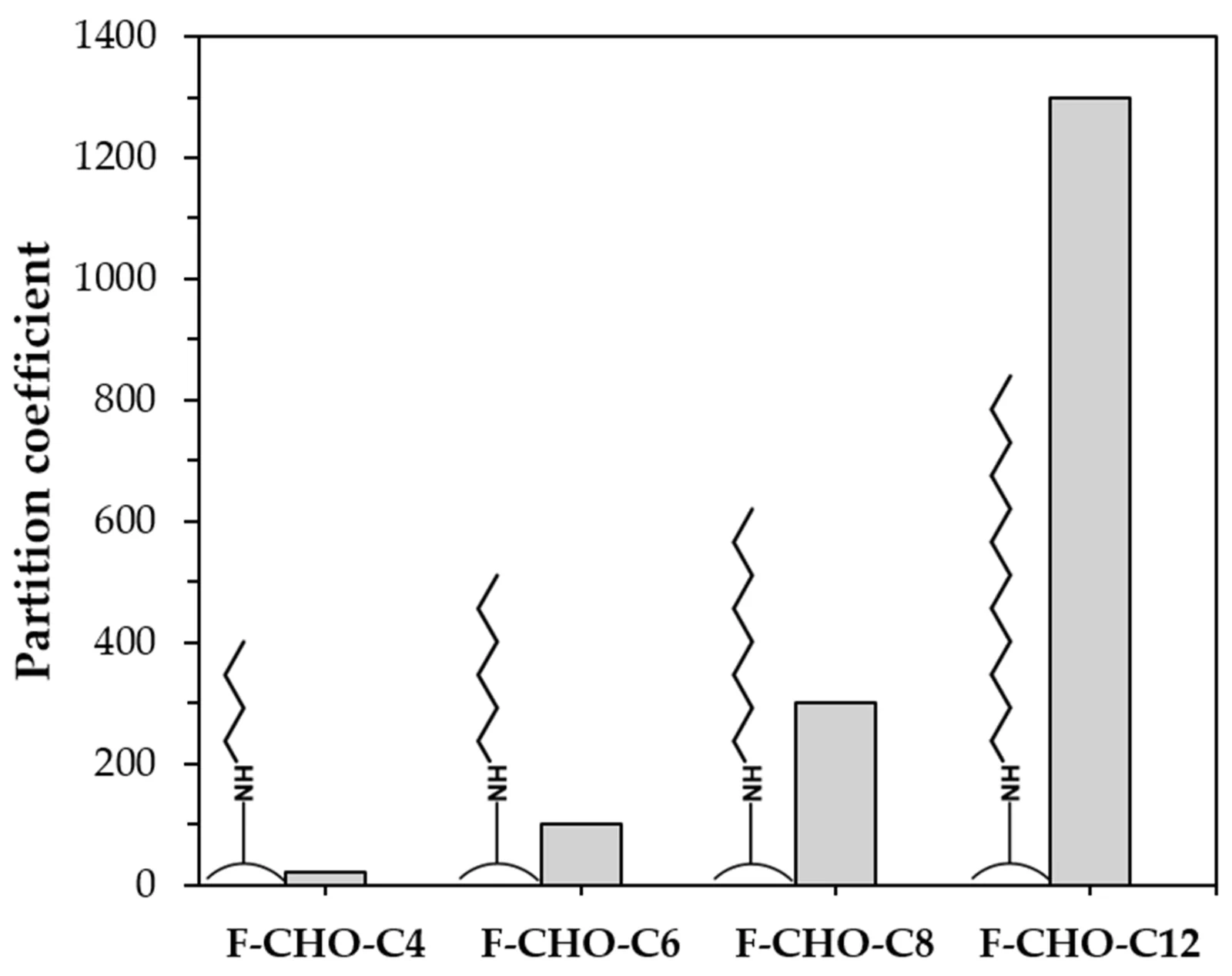
Partition coefficients of eosin Y predicted by COSMO-SAC.

As described above, the adsorption amount of the hydrophobic drug models per mole of carbon atoms in hydrophobic groups incorporated into gelatin was higher for linear fatty groups than for aromatic groups. For linear fatty groups, the adsorption amount increased as the carbon number increased. This point was further discussed using COSMO-SAC (thermodynamic calculations) and Gaussian (quantum chemical calculations) software. In the COSMO-SAC framework, the hydrophobic group phase in the hydrogel was represented using the corresponding fatty groups used for modification. Figure 9 shows COSMO-SAC calculation results of the partition coefficients of a hydrophobic drug model. In this model, the partition coefficient serves as a model-based affinity descriptor, where a higher value indicates that the hydrophobic drug partitions more favorably into the hydrophobic group phase of HMG than into the bulk water phase within the hydrogel. The figure shows that, as the carbon number of the linear fatty groups increased, the partition of the hydrophobic drug models into the hydrophobic groups increased exponentially. These calculation results largely support the experimental findings shown in Figure 7.

**Figure 10:**
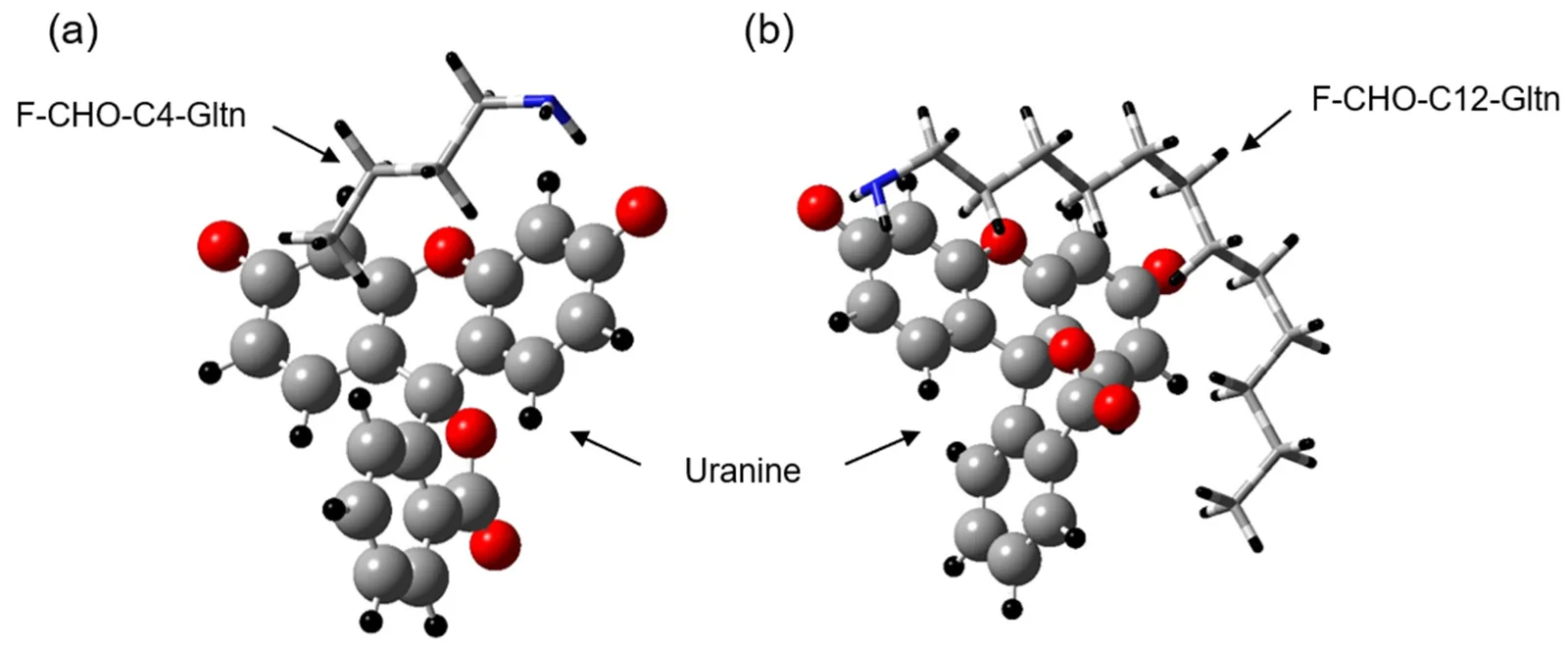
Results of geometry optimization for the F-CHO-C4/uranine (**a**) and F-CHO-C12/uranine (**b**) complexes during adsorption.

Next, to discuss the adsorption behavior at the molecular level, quantum chemical calculations were performed using the Gaussian software. In the quantum chemical calculations, the most stable structures of the F-CHO-C4/uranine and F-CHO-C12/uranine complexes were obtained by geometry optimization, and their interaction energies were calculated. In the calculations, the interaction energy between F-CHO-C12 and uranine was larger than that between F-CHO-C4 and uranine (Table 2). This is presumably because, as shown in the most stable structure in Figure 10, uranine is surrounded by the longer alkyl chain of F-CHO-C12 and interacts with it at multiple sites. The higher interaction energy resulting from the drug/longer-alkyl-chain complex supports the experimental finding that, as the carbon number of the linear fatty groups increases, the adsorption amount increases. Moreover, these calculation results suggest that, in the comparison between linear fatty and aromatic groups (F-CHO-C11 vs. A-CHO-C11), the linear fatty group can adsorb a larger amount of drug models than the aromatic group because it interacts with the drug models in such a manner that it surrounds them.

### 2.4. Release of Hydrophobic Drug Models from Unmodified Gelatin and HMG Hydrogel Particles

**Figure 11:**
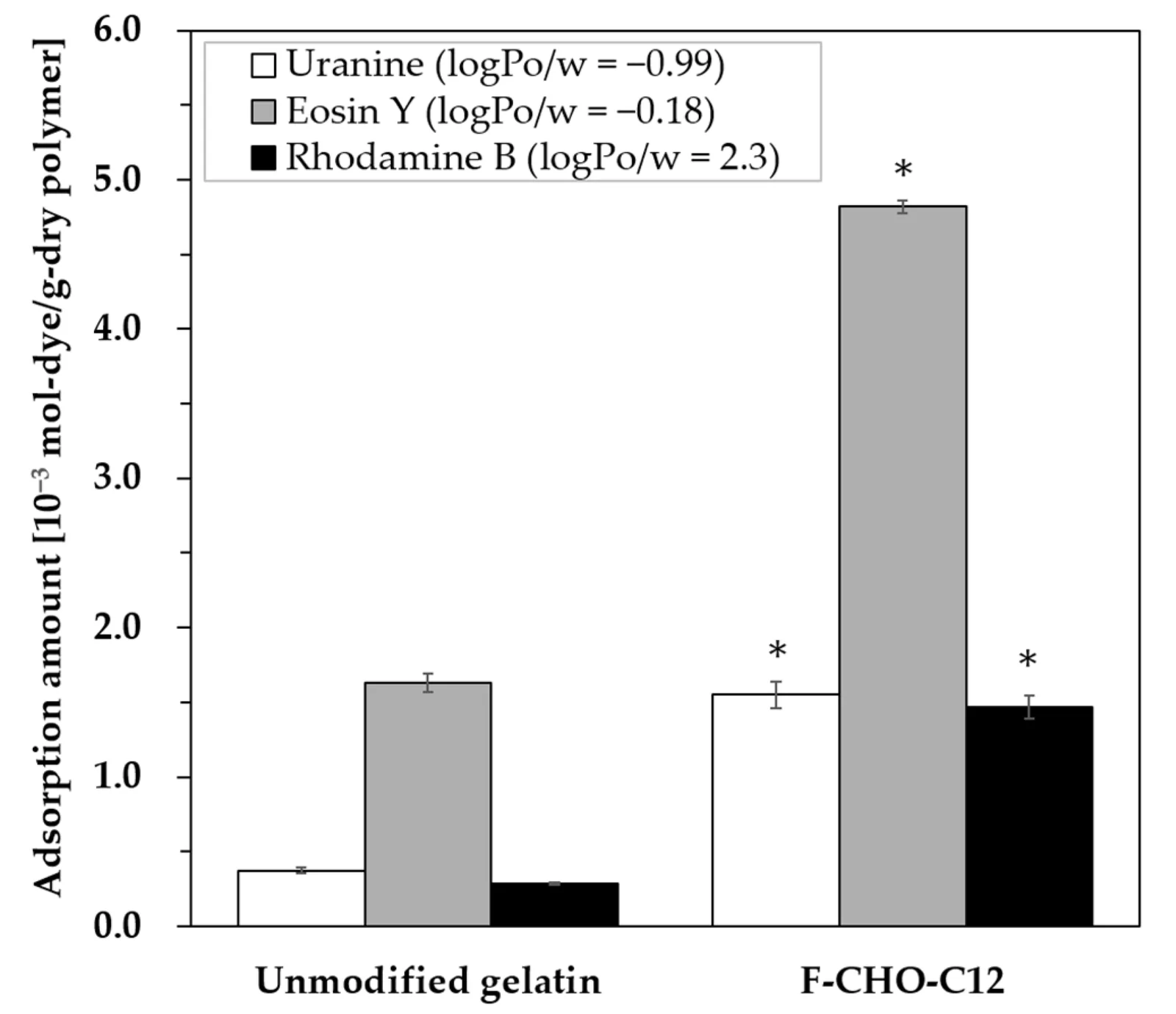
Adsorption amount of drug models per unit dry weight of unmodified gelatin and HMG with F-CHO-C12 at 4 °C (*n* = 3). * *p* < 0.05 vs. unmodified gelatin.

**Figure 12:**
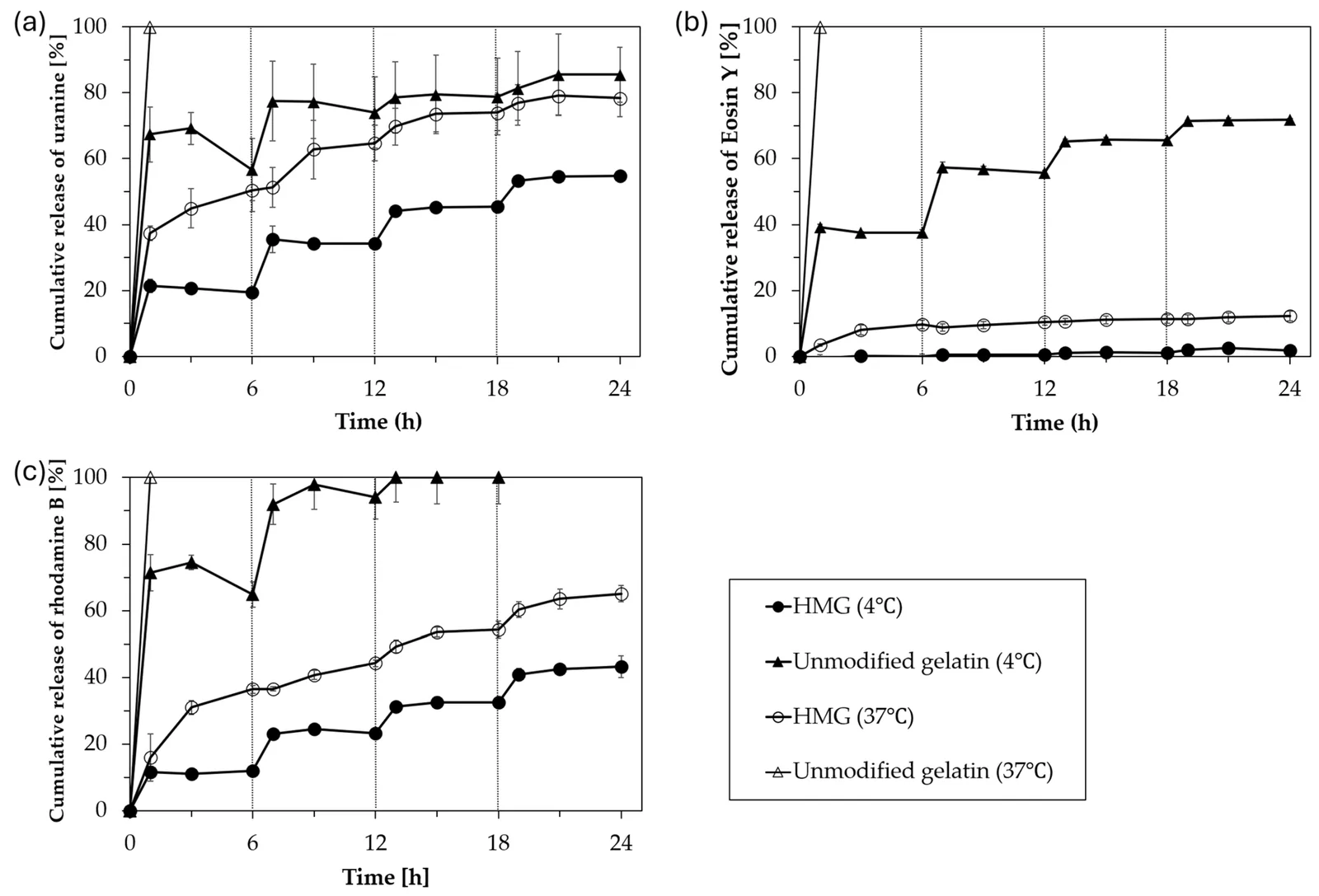
Release profiles of uranine (**a**), eosin Y (**b**) and rhodamine B (**c**) from unmodified gelatin and HMG with F-CHO-C12 in PBS(-) at 4 and 37 °C (*n* = 3). PBS(-) was exchanged for fresh PBS(-) every 6 h.

In Section 2.2, we compared the adsorption amount of the drug models per mole of carbon atoms in the hydrophobic groups incorporated into gelatin. On the other hand, it is also important to determine the adsorption amount per unit dry weight of the polymer. Therefore, we compared the adsorption amount per unit dry weight of the hydrogel particles of unmodified gelatin and HMG. As the hydrophobic group, we selected F-CHO-C12 because it is one of the hydrophobic groups with the highest adsorption performance. Hydrogel particles <1 mm in diameter (dry weight: 0.02 g) were shaken in a dye solution for 24 h to reach adsorption equilibrium. As shown in Figure 11, the adsorption capacity of the HMG per unit dry weight was higher than that of unmodified gelatin. It should be noted that, because unmodified gelatin was not incorporated with any hydrophobic groups, it is not possible to calculate the adsorption amount per mole of carbon atoms of incorporated hydrophobic groups. Next, for release of drug models, HMG hydrogel particles (0.095 g of dry polymer, in which the total number of moles of carbon atoms in the incorporated hydrophobic groups was 3.75 × 10^−4^ mol) and unmodified gelatin hydrogel particles with the same dry weight were added to aqueous solutions containing the drug models and shaken at 4 °C for 24 h to reach adsorption equilibrium. The differences in the release behavior were evaluated at both 4 °C and physiological temperature (37 °C) (Figure 12). An initial burst was observed for the unmodified gelatin at 4 °C. This is because the hydrophobic interactions between the drug models and gelatin are weak. On the other hand, HMG at 4 °C releases the drug models more slowly than unmodified gelatin. At 37 °C, the unmodified gelatin completely dissolved in the dye solution within 3 h. On the other hand, for HMG at 37 °C, although the release rate of the drug models was higher than that at 4 °C, the dramatic initial burst observed for unmodified gelatin was avoided. These findings suggest that HMG with F-CHO-C12 is suitable as a carrier material for the sustained release of hydrophobic substances. 

### 2.5. Adsorption/Release of Dexamethasone to/from Unmodified Gelatin and HMG Hydrogel Particles

**Figure 13:**
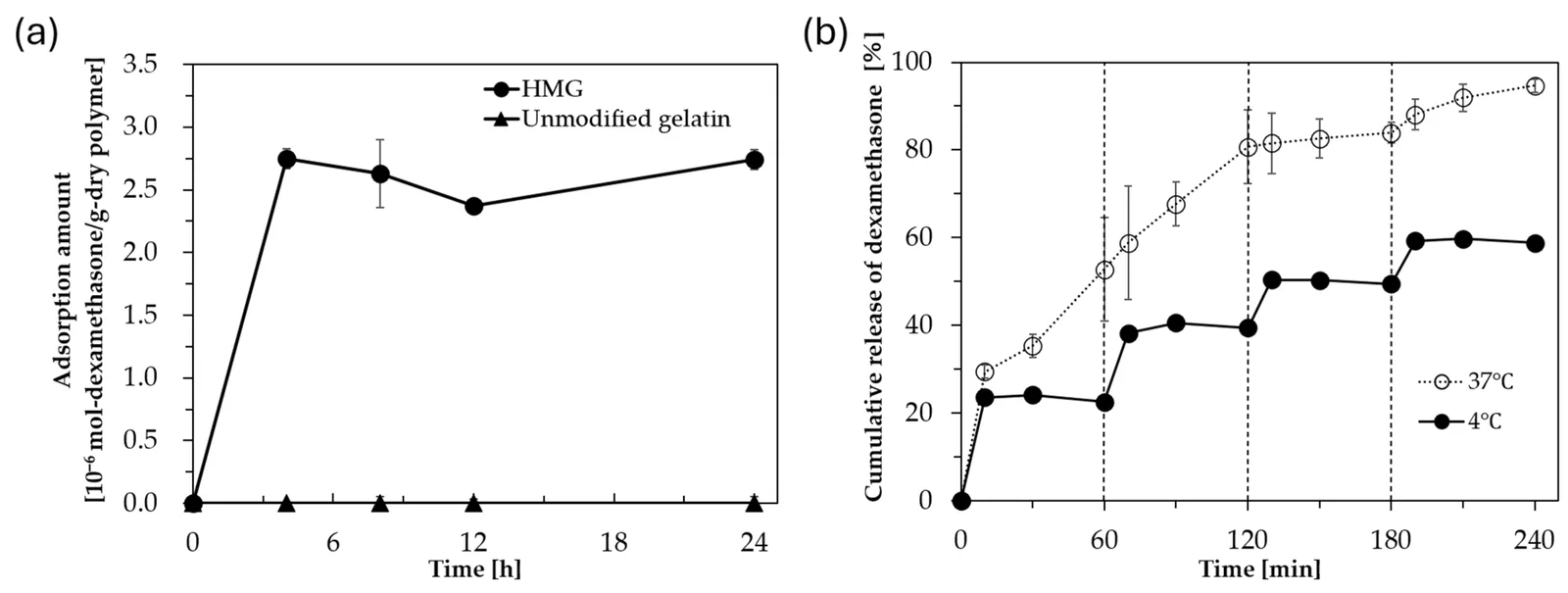
(**a**) Adsorption of dexamethasone on unmodified gelatin and HMG with F-CHO-C12 at 4 °C (*n* = 3). (**b**) Release profile of dexamethasone from HMG with F-CHO-C12 in PBS(-) at 4 and 37 °C. PBS(-) was exchanged for fresh PBS(-) every 60 min (*n* = 3).

In this study, dexamethasone (Log P_o/w_ = 1.83) was selected as a hydrophobic drug. Dexamethasone is used as an anti-inflammatory drug in the treatment of ocular diseases and ulcerative colitis, and it also has the ability to enhance bone formation [29,30]. However, dexamethasone can cause adverse effects such as osteoporosis, hyperglycemia, hypertension, and peptic ulcers [29]. To mitigate these adverse effects, attempts have been made to achieve sustained release of dexamethasone in vivo from polymeric microparticles [30,31,32]. Therefore, to evaluate the potential of HMG with F-CHO-C12 as a carrier for dexamethasone, we investigated the adsorption and release behavior of the drug to/from the hydrogel particles. HMG hydrogel particles, in which the incorporated hydrophobic groups corresponded to 7.5 × 10^−5^ mol (dry weight: 0.019 g), and an equal weight of unmodified gelatin hydrogel microparticles were added to 4 mL of an aqueous dexamethasone solution and shaken at 4 °C until adsorption equilibrium was reached. Unmodified gelatin exhibited negligible dexamethasone adsorption, as the decrease in absorbance was below the detection limit of the spectrophotometer (<$0.08\times 10^{-6}$ mol-dexamethasone/g-dry polymer), making it unfeasible to determine its exact loading amount and cumulative release profile (Figure 13a). In contrast, HMG achieved a substantial dexamethasone adsorption capacity of approximately 2.8 $\times \;10^{-6}$ mol-dexamethasone/g-dry polymer. Next, for release of dexamethasone, HMG hydrogel particles (dry weight: 0.095 g) were added to 4 mL of an aqueous dexamethasone solution and allowed to adsorb at 4 °C for 24 h. Subsequently, the hydrogel particles were transferred into PBS(-) and shaken at 4 °C or 37 °C. The cumulative release profile was evaluated relative to this experimentally loaded amount. At 4 °C, a stepwise release profile of dexamethasone from the HMG hydrogel particles was observed (Figure 13b). At 37 °C, the release rate was higher than that at 4 °C, reaching approximately 95% cumulative release without an uncontrolled initial burst. Because unmodified gelatin fails to show detectable dexamethasone adsorption, hydrophobic modification with F-CHO-C12 plays an essential role in conferring drug-binding capacity to gelatin and enabling sustained release at physiological temperature while maintaining hydrogel structural integrity.

It is worth noting that although rhodamine B has a higher Log P_o/w_ (2.30) than Eosin Y (−0.18), Eosin Y consistently exhibited higher adsorption amounts across the HMG hydrogels (Figure 7 and Figure 8). This indicates that the adsorption behavior within HMG hydrogels is governed not merely by the bulk hydrophobic partition coefficient (Log P_o/w_) but also by molecular geometry, hydrogen bonding, electrostatic interactions, and the conformational freedom of the incorporated hydrophobic groups. Eosin Y possesses multiple bromine atoms and phenolic/carboxyl hydroxyl groups, which facilitate strong hydrogen bonding and multi-site interactions with the gelatin backbone and modified sites. In contrast, rhodamine B features bulky diethylamino groups that induce significant steric hindrance when approaching the hydrophobic domains. Furthermore, the secondary amine anchor formed via reductive amination of aldehydes possesses single-bond rotational flexibility (high conformational freedom), allowing the alkyl chains to flexibly wrap around Eosin Y molecules—a phenomenon supported by our Gaussian calculations (Figure 10). Conversely, the amide anchor formed by acyl chlorides is more rigid due to its planar resonance structure, restricting such conformational adaptation.

The adsorption and desorption experiments in this study were conducted in PBS (pH 7.4). The increase in ionic strength has been shown to suppress electrostatic interactions and relatively enhance the contribution of hydrophobic interactions in multiple adsorption systems and theoretical studies [33,34,35,36,37]. However, the underlying mechanisms may vary depending on the system, and diverse factors such as electric double-layer screening, solvation entropy, and ion-specific effects are involved.

## 3. Conclusions

In this study, HMG hydrogels incorporating twelve distinct hydrophobic group structures were synthesized to systematically elucidate the influence of hydrophobic group structure, carbon number, and modification linkage on the loading and release behavior of hydrophobic model compounds. Under the evaluation criterion of adsorption capacity per mole of incorporated carbon atoms, linear fatty aldehydes—specifically, F-CHO-C11 and F-CHO-C12—exhibited the highest adsorption among the evaluated structures. The experimental comparisons and theoretical analyses provided the following structure–property insights: (1) linear fatty aldehyde modifications yielded higher adsorption capacities than linear fatty acyl chloride modifications, which is hypothesized to result from the absence of hydrophilic carbonyl linkages at the modified sites; (2) linear fatty side chains showed higher adsorption capacities than polycyclic aromatic groups, potentially attributable to lower steric hindrance and a reduced propensity for self-aggregation compared with bulky aromatics such as anthracene; and (3) increasing alkyl chain length enhanced drug loading efficiency per carbon atom. Thermodynamic calculations (COSMO-SAC) and quantum chemical simulations (Gaussian MP2) supported these observed trends, suggesting that longer alkyl chains facilitate multi-site interactions by encapsulating hydrophobic molecules. Furthermore, F-CHO-C12 HMG hydrogel exhibited substantial loading capacity for dexamethasone—a compound poorly loaded by unmodified gelatin—and effectively reduced initial burst release at 37 °C during release testing. Overall, these findings offer useful design guidelines for hydrophobically modified polymers and demonstrate that F-CHO-C12 HMG serves as a promising proof-of-concept sustained-release carrier for hydrophobic drugs.

## 4. Materials and Methods

### 4.1. Materials

Gelatin from bovine skin (type B) was purchased from Sigma-Aldrich Co. (St. Louis, MO, USA). Linear fatty aldehydes (butanal (F-CHO-C4), hexanal (F-CHO-C6), heptanal (F-CHO-C7), octanal (F-CHO-C8), undecanal (F-CHO-C11) and dodecanal (F-CHO-C12)), benzaldehyde (A-CHO-C7), 1-naphthaldehyde (A-CHO-C11), 9-anthracenecarbaldehyde (A-CHO-C15), dimethyl sulfoxide (DMSO), triethylamine (TEA), uranine and eosin Y were obtained from Wako Pure Chemical Industries, Ltd. (Osaka, Japan). Linear fatty acyl chlorides (octanoyl chloride (F-COCl-C8) and palmitoyl chloride (F-COCl-C16)), phenylacetyl chloride (A-COCl-C8), rhodamine B and dexamethasone were obtained from Tokyo Chemical Industry, Co., Ltd. (Tokyo, Japan). 2-methylpyridine borane was obtained from Junsei Chemical Co., Ltd. (Tokyo, Japan).

### 4.2. Synthesis of HMG

HMG incorporated with either linear fatty aldehydes (F-CHO-C4, C6, C7, C8, C11 or C12) or aromatic aldehydes (A-CHO-C7, C11 or C15) were synthesized according to our previous reports (Figure S1) [21,26]. Briefly, the gelatin was dissolved in 100 mL of PBS(-) (pH 7.4) at a concentration of 3% (*w*/*v*) by heating. Ethanol (99.8% (*v*/*v*)) was added to the solution at a volume ratio of 1:1 at room temperature. Linear fatty aldehyde or aromatic aldehydes dissolved in a small volume of ethanol were added to the gelatin solution and stirred for 6 h at room temperature to form imine bonds between the aldehyde groups and the amino group of the gelatin. Table 1 lists the feeding molar ratios of linear fatty aldehydes or aromatic aldehydes to free amino groups of gelatin. A reductant, 2-methylpyridine borane was dissolved in the solution at a feeding molar ratio of 300% to the free amino groups and stirred for 24 h for reductive amination. A large amount of ethanol was added to the aqueous solution to precipitate the HMG incorporated with the hydrophobic groups. The precipitates were frozen in liquid nitrogen and crushed to obtain particles. The frozen particles were vacuum-dried to obtain dried HMG particles <1 mm in diameter. In the case of A-CHO-C15, HMG was synthesized using the same procedure as described above, except that the gelatin concentration in PBS(-) was changed to 1% (*w*/*v*).

The HMG incorporated with either linear fatty or aromatic acyl chlorides (F-COCl-C8, C16 or A-COCl-C8) was synthesized according to the literature reported by Mizuta et al. with some modifications (Figure S2) [27]. The gelatin was first dissolved in DMSO at a concentration of 2.5% (*w*/*v*) by heating. TEA was added to the solution in a feeding molar ratio of 1:1 to free amino groups of the gelatin. Either linear fatty or aromatic acyl chloride dissolved in a small amount of DMSO was added to the gelatin solution and stirred overnight at 50 °C under a nitrogen atmosphere (Table 1). Dried particles (<1 mm in diameter) of the HMG were obtained through precipitation by dialysis in a large amount of ethanol, collected by centrifugation, and lyophilized. 

The incorporation of hydrophobic groups into gelatin was qualitatively confirmed using FT-IR (IRT-3000, JASCO Co., Ltd., Tokyo, Japan). The degree of substitution of hydrophobic groups was estimated by determining the number of unreacted amino groups in HMG using trinitrobenzene sulfonic acid (TNBS) assay [27]. Because both the anthracene moiety of A-CHO-C15 and TNBS absorb at 350 nm, the degree of substitution for A-CHO-C15 was estimated using the ninhydrin method.

### 4.3. Swelling Ratio of HMG

Unmodified gelatin and HMG dry particles were placed in centrifuge tubes such that the dry polymer weight ($W_{dry}$) was 0.05 g and immersed in 2 mL of PBS(-). After incubation for 12, 24, and 48 h at 4 °C, the samples were centrifuged, and the supernatant was removed. The combined weight of the swollen polymer and the centrifuge tube was measured, and $W_{wet}$ was obtained by subtracting the premeasured tube weight. The swelling ratio was calculated as following equation.

(1) $$Swelling\;ratio\;[\%]=\frac{W_{wet}-W_{dry}}{W_{dry}}\times 100$$

where $W_{wet}$ and $W_{dry}$ are the weights of the swollen and dry polymers, respectively.

### 4.4. Adsorption/Release of Hydrophobic Drug Models and Dexamethasone to/from Unmodified Gelatin and HMG Hydrogel Particles

For adsorption of drug models and dexamethasone on unmodified gelatin and HMG hydrogel particles <1 mm in diameter, the hydrogel particles were first prepared by immersing dried particles in ethanol, then in PBS(-), at 4 °C. Ethanol was used to make the hydrophobic particles wet with PBS(-). The morphology of the hydrogel particles was observed using a phase microscope (Leica DM IL LED, Leica Microsystems, Wetzlar, Germany). Three types of dyes with different hydrophobicities as models of hydrophobic drugs were used (Figure 2). The unmodified gelatin and the HMG hydrogel particles were gently shaken in 4 mL of PBS(-) with dissolved dyes or dexamethasone (either 25 µM uranine, 25 µM eosin Y, 15 µM rhodamine B or 40 µM dexamethasone) at 4 °C. At predetermined intervals, the amounts of the dyes and dexamethasone in the solution were determined by measuring the absorbance of the PBS(-) (uranine: 490 nm; eosin Y: 517 nm; rhodamine B: 554 nm; dexamethasone: 242 nm). The equilibrium adsorption amount was calculated based on the absorbance.

For release from HMG hydrogel particles, unmodified gelatin and HMG hydrogel particles were added to 4 mL of a dye or dexamethasone solution (either 50 µM uranine, 50 µM eosin Y, 35 µM rhodamine B or 65 µM dexamethasone) and gently stirred for 24 h at 4 °C to reach adsorption equilibrium. The hydrogel particles were then transferred to 4 mL of PBS(-) at 4 °C or 37 °C and shaken. The PBS(-) was exchanged for fresh PBS(-) every 6 h. The amount of drug models and dexamethasone released from the hydrogel particles was determined by measuring the absorbance of the PBS(-). The release amount (Q) was calculated using the following equation.

(2) $$Q=\frac{C\times V}{q}\times 100$$

where C is the drug concentration in the solution, V is the solution volume, and q is the equilibrium adsorption amount. After each PBS replacement, the cumulative release amount was calculated by adding the amount released before the replacement to the amount released after the replacement.

### 4.5. Prediction of Octanol–Water Partition Coefficients of Hydrophobic Groups Incorporated into Gelatin Using ChemDraw

The hydrophobic groups were assumed to react with NH_2_–CH_3_ (as the most simple model for gelatin). The octanol–water partition coefficients of the resulting products were calculated using ChemDraw Professional (Version 23.1.2, PerkinElmer Informatics).

### 4.6. Prediction of Partition Coefficient of Drug Models Using COSMO-SAC

Molecular affinities of hydrophobic drugs on the gelatin incorporated with hydrophobic groups are important information for the design of the HMG. A partition coefficient ($K_{d}$) of the hydrophobic drug between hydrophobic groups incorporated into gelatin and water in hydrogel can be used for the evaluation of the molecular affinities of the hydrophobic drug to the HMG.

(3) $$K_{d}=\frac{\gamma_{d}^{W}}{\gamma_{d}^{H}}$$

where $\gamma_{d}$ is the activity coefficient of the hydrophobic drug and the superscripts H and W represent the hydrophobic group and water phases, respectively. The activity coefficients of the hydrophobic drug are calculated by the COSMO-SAC method as shown in the following equation [22]. COSMO quantum calculation was conducted using BIOVIA TmoleX 2025 version 25.0.0 based on basis function def-TZVP and DFT setting BP86-m3. Molecular surface charge densities can be obtained from this COSMO quantum calculation, and that information was installed into the activity coefficient calculation based on COSMO-SAC. The COSMO-SAC parameter in the original literature [22] was used in these calculations. The partition coefficient of the hydrophobic groups in the hydrogel was calculated based on the fatty acid molecules used for the hydrogel modification.

(4) $$ln\gamma_{d}=ln\gamma_{d}^{C}+\frac{A_{d}}{a_{seg}}\sum_{\sigma }p(\sigma )\left[ln\Gamma \left(\sigma \right)-ln\Gamma_{d}\left(\sigma \right)\right]$$

drug molecule and the segment on the molecular surface. Γ(σ) and p(σ) are the segment activity coefficient and molecular surface charge profile with a charge of σ.

### 4.7. Estimation of Adsorption Behavior Using the Gaussian Software

Geometry optimizations were performed for the F-CHO-C4/uranine and F-CHO-C12/uranine complexes, as well as for their corresponding isolated molecules, to determine the most stable structures. All calculations were carried out at the MP2/6-31G(d) level of theory, with the effect of aqueous solvation via the polarizable continuum model. These calculations were performed using Gaussian 16 [38], and initial molecular structures were prepared using GaussView 6 [39]. Interaction energies were calculated as the difference between the total energy of the complex and the sum of the total energies of the corresponding isolated molecules. Basis set superposition error corrections were not applied.

### 4.8. Statistical Analysis

Data are presented as mean ± standard deviation. Statistical differences between two groups were analyzed using Student’s *t* test. Statistical differences among more than three groups were analyzed using one-way analysis of variance (ANOVA) with Tukey analysis.

## Figures and Tables

**Table 1:** Degree of substitution of hydrophobic groups to gelatin.

Name	Carbon Number of Hydrophobic Groups	Terminal Reactive Group of Hydrophobic Groups	Feeding Molar Ratios of Hydrophobic Groups to Amino Groups of Gelatin (%)	Degree of Substitution (%)
Unmodified gelatin	-	-	-	-
F-CHO-C4	C4	Aldehyde	77	43
F-CHO-C6	C6	Aldehyde	77	38
F-CHO-C7	C7	Aldehyde	77	45
A-CHO-C7	C7	Aldehyde	154	42
F-CHO-C8	C8	Aldehyde	77	42
F-COCl-C8	C8	Acyl chloride	41	44
A-COCl-C8	C8	Acyl chloride	184	50
F-CHO-C11	C11	Aldehyde	77	38
A-CHO-C11	C11	Aldehyde	154	50
F-CHO-C12	C12	Aldehyde	77	41
A-CHO-C15	C15	Aldehyde	154	36
F-COCl-C16	C16	Acyl chloride	205	46

**Table 2:** MP2 total energies and interaction energies of hydrophobic group/uranine complexes.

Hydrophobic Group	E_Uranine_ (Hartree)	E_Hydrophobic group_ (Hartree)	E_complex_ (Hartree)	ΔE_interaction_ (kcal/mol)
F-CHO-C4	−1141.155954	−213.011883	−1354.176961	−5.73
F-CHO-C12	−1141.155954	−526.337917	−1667.512667	−11.8

## Data Availability

Data presented in this study are available on request from the corresponding authors.

## Supplementary Materials

The following supporting information can be downloaded at https://www.mdpi.com/article/10.3390/gels12100941/s1, Figure S1: Chemical reaction to incorporate hydrophobic groups possessing aldehyde groups into gelatin; Figure S2: Chemical reaction to incorporate hydrophobic groups possessing acyl chloride groups into gelatin; Figure S3: Photos of unmodified gelatin (a, n) and HMG microparticles prepared using F-CHO-C4 (b, o), F-CHO-C6 (c, p), F-CHO-C7 (d, q), A-CHO-C7 (e, r), F-CHO-C8 (f, s), F-COCl-C8 (g, t), A-COCl-C8 (h, u), F-CHO-C11 (i, v), A-CHO-C11 (j, w), F-CHO-C12 (k, x), A-CHO-C15 (l, y), F-COCl-C16 (m, z) before (a-m) and after (n-z) swelling in PBS(-); Figure S4: Schematic diagram for comparing drug model adsorption under equivalent molar carbon atom conditions using hydrophobic groups with different carbon numbers.
