# Supplementary material for: Optimization of Hydrophobic Group Structures Covalently Incorporated into Gelatin Hydrogels for Efficient Adsorption and Release of Hydrophobic Drugs
Source: Gels. 2026 Oct 21;12(10):941. doi: 10.3390/gels12100941 (PMC13704556; doi:10.3390/gels12100941)
Supplement: Supplementary file 1 [file gels-12-00941-s001.zip › gels-4560546-supplementary.pdf]

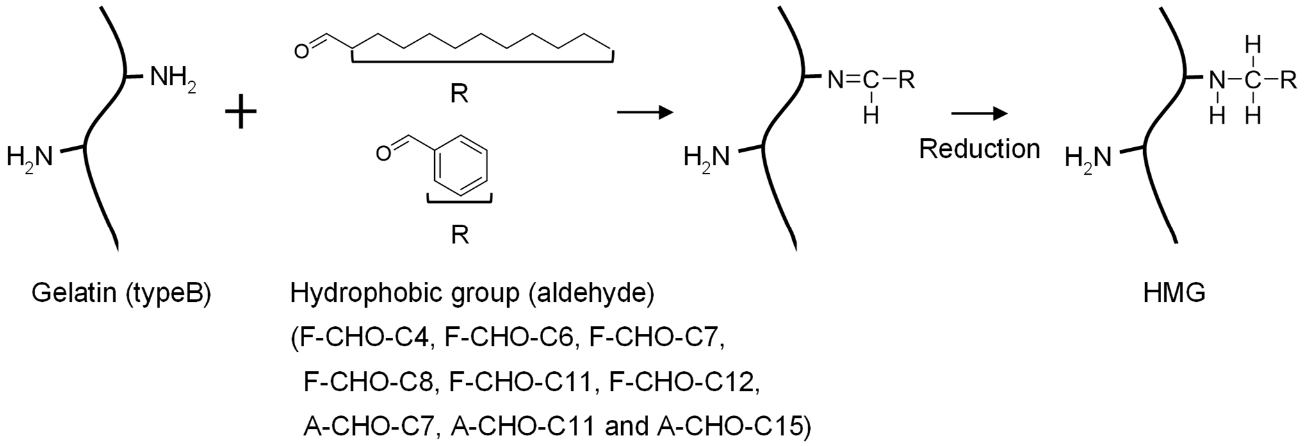

Figure S1. Chemical reaction to incorporate hydrophobic groups possessing aldehyde groups into gelatin.

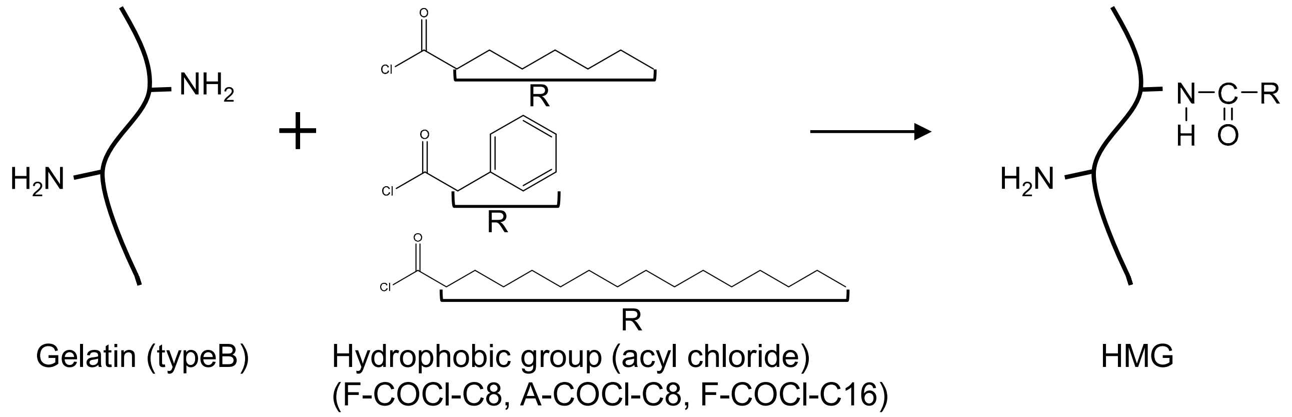

Figure S2. Chemical reaction to incorporate hydrophobic groups possessing acyl chloride groups into gelatin.

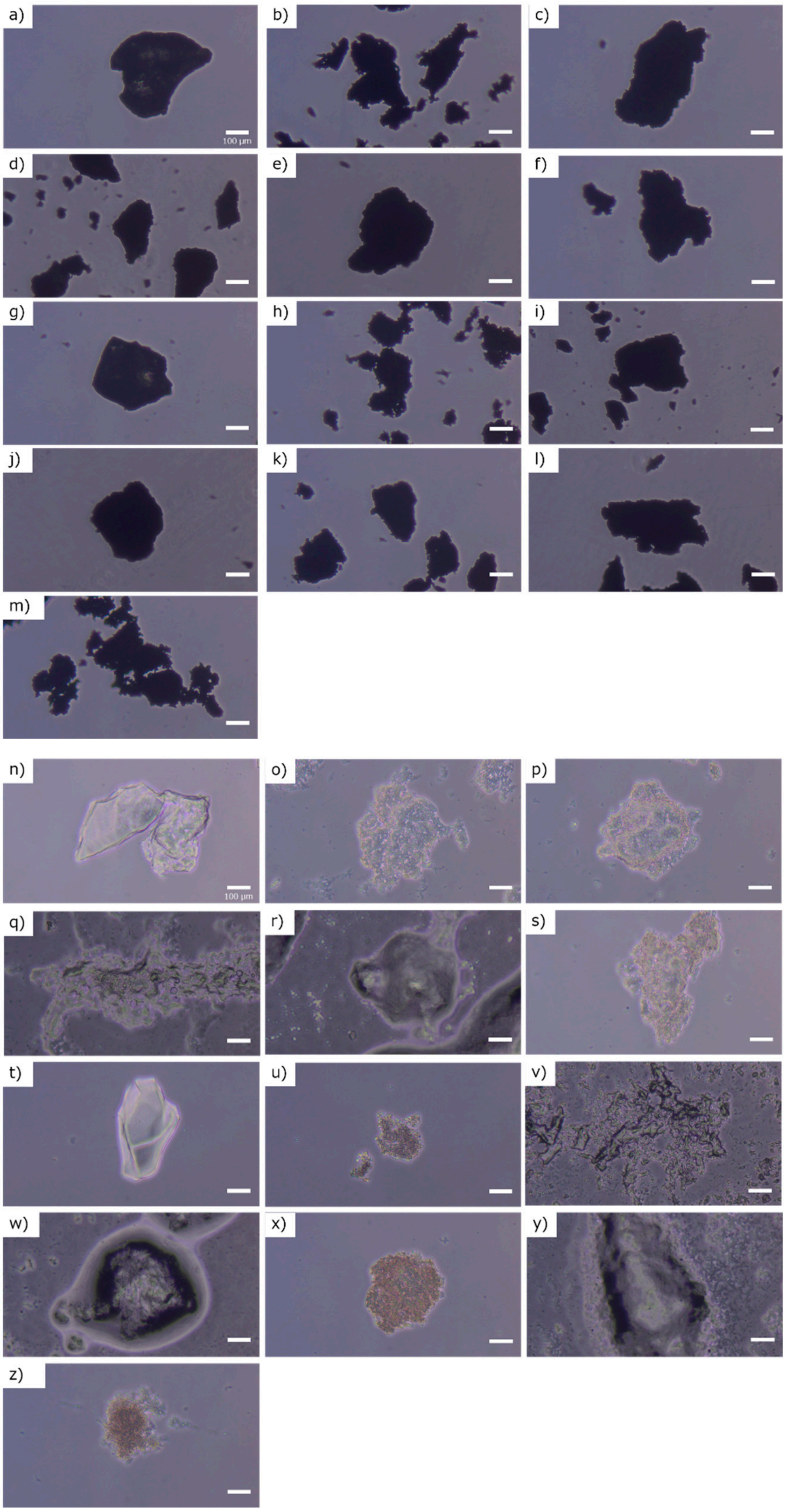

Figure S3. Photos of unmodified gelatin (a, n) and HMG microparticles prepared using F-CHO-C4 (b, o), F-CHO-C6 (c, p), F-CHO-C7 (d, q), A-CHO-C7 (e, r), F-CHO-C8 (f, s), F-COCl-C8 (g, t), A-COCl-C8 (h, u), F-CHO-C11 (i, v), A-CHO-C11 (j, w), F-CHO-C12 (k, x), A-CHO-C15 (l, y), F-COCl-C16 (m, z) before (a-m) and after (n-z) swelling in PBS(-).

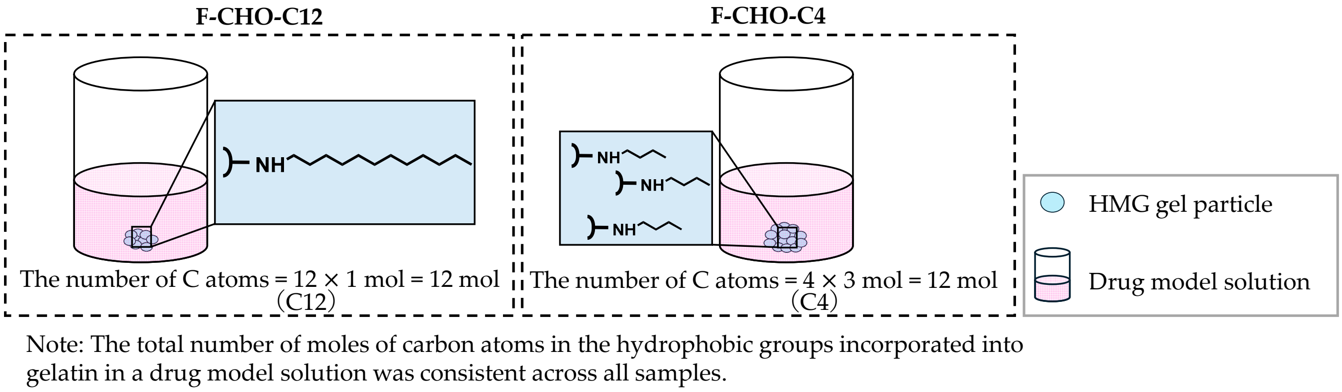

Figure S4. Schematic diagram for comparing drug model adsorption under equivalent molar carbon atom conditions using hydrophobic groups with different carbon numbers.
